# Intricacies of Radiographic Assessment in Testicular Germ Cell Tumors

**DOI:** 10.3389/fonc.2020.587523

**Published:** 2021-01-05

**Authors:** Marek Makovník, Katarína Rejleková, Ivan Uhrin, Michal Mego, Michal Chovanec

**Affiliations:** ^1^ Radiology Department, National Cancer Institute, Bratislava, Slovakia; ^2^ 2nd Department of Oncology, Faculty of Medicine, Comenius University and National Cancer Institute, Bratislava, Slovakia; ^3^ Translational Research Unit, Faculty of Medicine, Comenius University, Bratislava, Slovakia

**Keywords:** follow-up, post-chemotherapy, active surveillance, magnetic resonance, computed tomography, testicular germ cell tumors, testis, positron emission tomography/computer tomography (PET/CT)

## Abstract

Testicular germ cell tumors (GCTs) are malignancies with a unique biology, pathology, clinical appearance, and excellent outcomes. A correct radiographic assessment of GCTs is extremely important for the clinical management in several typical scenarios. Advancements in the field of diagnostic medicine bring an increasing number of sophisticated imaging methods to increase the performance of imaging studies. The conventional computed tomography (CT) remains the mainstay of diagnostic imaging in the management of GCTs. While certain improvements in the sensitivity and specificity are suggested with magnetic resonance (MR) imaging with lymphotrophic nanoparticles in evaluating retroperitoneal lymph nodes during the staging procedure, further exploration in larger prospective studies is needed. A common diagnostic dilemma is assessing the post-chemotherapy residual disease in GCTs. Several studies have consistently shown advantages in the utility of positron emission tomography (PET) scanning in post-chemotherapy residual retroperitoneal lymph nodes in patients with seminoma, but not with non-seminoma. Recommendations suggest that seminoma patients with a residual disease in the retroperitoneum larger than 3 cm should be subjected for PET scanning with 18-fluorodeoxyglucose. Relatively high sensitivity, specificity and a negative predictive value (80–95%) may guide clinical decision to spare these patients of high morbidity of an unnecessary surgery. However, a positive predictive value of around 50% renders PET scanning difficult to interpret in the case of positive finding. These patients often require extremely difficult surgical procedures with the high risk of post-operative morbidity. Therefore, seminoma patients with PET positive residual masses larger than 3 cm still remain a serious challenge in the decision making of nuclear medicine specialist, oncologists, and urologic surgeons. In this article, we aim to summarize data on controversial dilemmas in staging procedures, active surveillance, and post-chemotherapy assessment of GCTs based on the available published literature.

## Introduction

Testicular germ cell tumors (GCTs) are the most common type of non-hematologic malignancy in males from the ages of 15 to 49. GCTs account for 1% of all male neoplasm (1). Cryptorchidism, radiation, undescended testis, testicular dysgenesis (testicular feminization, Klinefelter syndrome) and family history are considered to be the main risk factors that could lead to developing GCT (2). Owing to the exceptional sensitivity to cisplatin-based chemotherapy, GCTs are considered to be the only universally curable solid malignancy with the long‐term cure rate of more than 95% (3). The proper application of radiologic imaging and its correct interpretation by radiologist are essential and therefore play a crucial role for diagnostics, treatment response assessment, decision-making, and follow-up for testicular cancer patients. Computed tomography (CT) is used as a primary modality for imaging (4). Plain radiography, magnetic resonance (MR), and 18F-fluorodeoxyglucose positron emission tomography/computed tomography (FDG-PET/CT) are additional methods within the complex management of controversial dilemmas in staging procedures, active surveillance, and post-chemotherapy assessment of GCTs.

## Brief Overview of Staging and Risk-Stratification Principles in GCTs

**Figure 1:**
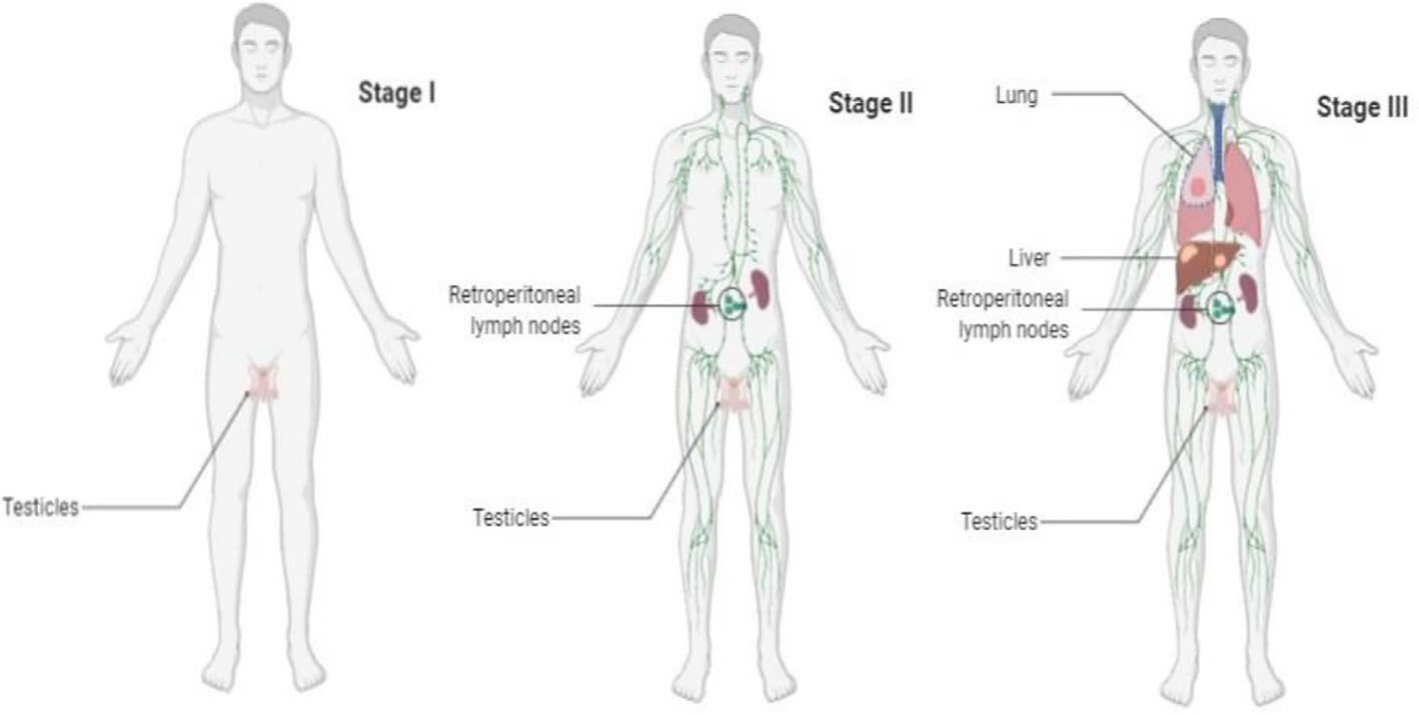
Graphical overview of GCT stage.

The mainstay in tailoring treatment of GCTs is a determination of clinical stage (**Figure 1**). Among factors to determine the clinical stage are the localization of primary tumor and/or the presence of metastases (5).

Stage I disease is characterized as tumor confined to the testicle with normal post-orchiectomy tumor markers alpha-fetoprotein (AFP), beta-human chorionic gonadotropin (HCG), lactate dehydrogenase (LDH), and absent metastases on CT scan. The majority of the patients with pure seminoma is diagnosed in stage I disease (80%). Tumor size (>4 cm) and invasion of rete testis are still considered negative prognostic factors for relapse; however, validation studies failed to confirm prognostic value of these risk factors (6, 7). Approximately 50% of patients with non-seminoma (NS) are diagnosed with stage I disease (8). Commonly accepted negative prognostic factors for relapse in stage I non-seminoma is invasion to the blood and lymphatic vessels and the presence of more than 50% of embryonic carcinoma (9, 10).

In stage II disease, patients have metastases in retroperitoneal (regional) lymph nodes, while patients in stage III disease have non-regional lymph node involvement and/or visceral metastases. The stage and prognosis is further refined with presence of elevated serum tumor markers. The International Germ Cell Cancer Collaborative Group (IGCCCG) has designed and established a risk-adapted classification dividing the patients with metastatic disease into three prognostic groups (11). The risk factors for the IGCCCG intermediate or poor risk disease are elevated levels of AFP, HCG, and LDH and the presence of non-pulmonary visceral metastases (6). The IGCCCG classification will soon be updated in the second version as a result of an international multi-institutional retrospective initiative (12).

## The Mode of Metastatic Spread

The key for understanding the metastatic process of GCTs lays in the human anatomy as well as biology of histological subtypes of GCTs. There are two accepted paths of metastasizing *via* haematogeneous or/and lymphatic spread. The knowledge of these patterns by the radiologist is essential for the correct imaging readings. The first site of the lymphatic spread are the retroperitoneal lymph nodes which can be followed by further spread into posterior mediastinum and other distant lymph nodes (5, 13, 14). However, the first site of lymphatic spread in case of a history of undescended testicle, vasectomy or inguinal herniorrhaphy is altered to the ipsilateral pelvic lymph-nodes (15). Retroperitoneal nodes have a specific laterality spread pattern. The inter-aortocaval lymph nodes and the right para-caval nodes are the most common landing zone from the right testicle. The left para-aortic lymph nodes (typically under the renal hilum) represents the most common landing zone from the left testicle (5, 13). The laterality of metastatic spread into retroperitoneal lymph nodes is an important factor in considering a virgin and post-chemotherapy retroperitoneal lymph node dissection (RPLND) templates (16). The role of imaging procedures and radiologist is to critically assess the retroperitoneal region for the suspicious lymph nodes. Such assessment is vital for the correct indication of unilateral modified versus bilateral template surgeries by an expert urologic surgeon in order to maintain an ejaculatory function (17).

The most common sites of haematogeneous metastases are lungs, followed by liver, brain, bone and other soft tissue loci. The pattern of developing metastases in lungs varies from peripherally located small multinodular lesions typical for non-seminomatous germ cell tumors (NSGCTs), to fewer larger looking lesions common in seminoma. Choriocarcinoma patients with a picture of military lung metastases and extremely high levels of HCG (> 100 000 mIU/ml) have a very poor prognosis after the initiation of treatment (18). Among the rare sites of metastatic spread are kidneys, adrenals, and spleen (5).

## The Role of Computed Tomography and Magnetic Resonance in Diagnostic Assessment of GCTs

### Computed Tomography and Magnetic Resonance in the Staging of Non-seminoma and Seminoma

Computed tomography (CT) remains the standard imaging method in the diagnosis and management of GCTs. The ability to capture the selected extent of body in one examination session during CT scanning allows for quick and efficient diagnosis. One of the major concerns is the radiation exposure in a young patient cohort. The excellent ability to predict the mode of spread in GCTs allows for a diagnostic accuracy with the standard computed tomography (CT) protocols, including the multiphase scanning of thorax, abdomen, and pelvis. The criteria for assessing the retroperitoneal lymph nodes are size and morphology. Some morphologic features can provide information on the histology type, such as the presence of a central necrosis in a normal soft tissue lymph node seen in seminoma, and large heterogeneous masses with cystic component can indicate possible NSGCTs (13). At the initial staging, computed tomography is able to detect 70–80% of positive retroperitoneal lymph nodes, while this can differentiate according to the cut off values of lymph node size (19). Several studies focused on lymph node size as a fulcrum that can predict the tumor infiltration. According to the study done by Hilton et al. there is a 37% sensitivity and 100% specificity after marking lymph nodes 10 mm or larger on CT as positive. Therefore a 10 mm cut off would miss over 60% of positive lymph nodes (20). A study by Hudolin et al. has shown that with a decrease in the cut-off value to 7–8 mm, a specificity and sensitivity of 70% can be achieved (21, 22). Further reduction of cut-off values would result in a significant reduction of specificity to 58%, which is undesirable despite the 90% sensitivity (20, 23). While the consensus is lacking, the recommended cut-off values for retroperitoneal lymph nodes are 8–10 mm (5, 13). According to the study by Cremerius et al. a sensitivity of 73% and specificity of 94% can be achieved with computed tomography at initial staging. These results were obtained from 50 patients with seminoma, embryonal carcinoma, teratocarcinoma, mixed tumor, and teratoma (23).

MRI is generally not recommended for staging and monitoring treatment response (13, 24). This is a common practice despite the similarity of diagnostic performance with CT scan when it comes to assessing the retroperitoneal lymph nodes. This thesis is supported by the findings of two studies that directly compared these two diagnostic tools. The first analyzed the retroperitoneal lymph nodes of 25 patients with NSGCT, finding that the MRI can predict the presence or absence of adenopathy in 84% of cases. The correct stage was assigned in 80% in contrast to the CT scanning which has shown the ability to predict the presence or absence of the disease in 88% and the correct stage was assigned in 84% (13). The second study was a prospective study of 52 patients (23 seminoma, 29 non-seminomatous or mixed germ cell tumors). Thirty-three patients were submitted for primary staging, ten patients underwent an evaluation following the treatment, and nine patients underwent the re-staging for a recurrent disease. Authors have found that the sensitivity for experienced reader between these two modalities is comparable and thus excluded the inferiority of MRI to the CT [94% (95%: CI 80–100%) to 98% (95%: CI 87–100%)] (25). Using the MRI, the radiologist is able to assess the retroperitoneal lymph nodes according to the morphology and size. However, a method called MR imaging with lymphotrophic nanoparticles (LNMRI) can effectively raise sensitivity and specificity in evaluating lymph nodes. The nanoparticles are trapped in the positive lymph nodes, thus changing the magnetic properties read by MRI (26–28). According to the study by Harisinghani et al., where 18 patients with stage I testicular cancer were analyzed (42 lymph nodes were sampled), authors came to the conclusion that sensitivity can be raised to 88.2% from 70.5%, and specificity can be raised to 92% from 68% compared to the plain MRI. Despite the promising results, the significant limitation of the LNMRI is the need for two separate scans done in 24 to 36 h apart (27, 28).

### Computed Tomography and Magnetic Resonance in the Post-Chemotherapy Management of Non-Seminoma and Seminoma

Post-chemotherapy residual disease often represents a radiologic dilemma due to the non-specific appearance of the residual tissue. As a result, the sensitivity and the negative predictive value are often low. A study assessing 85 residual lesions in patients with NSGCT has shown the sensitivity, specificity, positive predictive value, and negative predictive value of 55% (95% CI = 40 to 69%), 86% (95 CI = 79 to 100%), 84% (95 CI = 67 to 95%), and 58% (95% CI = 44 to 72%), respectively (29). A retrospective validation of the SEMPET trial analyzed 127 residual lesions in patients with seminoma. The sensitivity, specificity, negative predictive value, and positive predictive value were 67% (95% CI = 45 to 83%), 44% (95% CI = 35 to 54%), 87% (95% CI = 76 to 94%), and 19% (95% CI = 12 to 30%), respectively, for the residual disease regardless of the tumor size (30).

### Pulmonary Nodules

Small pulmonary nodules may represent another diagnostic dilemma during the initial staging. These nodules may represent metastases as well as benign lesions. A large study analyzing the incidental finding of small pulmonary nodules in GCTs is missing. Post-chemotherapy pulmonary lesions were analyzed in a retrospective study of 41 patients with NSGCT. One hundred and thirty-five metastatic pulmonary nodules after chemotherapy were assessed with the intention to find a cut-off value for predicting necrosis. All 135 pulmonary nodules were resected. Necrosis, teratoma, and viable cancer cells were found in 27 (65.9%; 95% CI, 49.4–79.9%), 12 (29.3%; 95% CI, 16.1–45.5%), and 2 (4.9%; 95% CI, 0.6–16.5%) lesions, respectively. The study showed the optimal long-diameter cut-off length for predicting necrosis to be 9 mm. Patients with teratoma components in the primary tumor were candidates for the immediate pulmonary resection, because of the high predicted risk of recurrence. Patients with the absence of teratoma components in the primary tumor and residual pulmonary nodules shorter than 10 mm after chemotherapy were associated with pathologic necrosis (31).

Treatment with bleomycin can induce a toxic pneumonitis resulting in the diffuse alveolar damage and pulmonary fibrosis. CT imaging may often show pulmonary nodules predominantly localized in the sub-pleural region of the lower third of lungs. Such lesions can mimic, should be carefully monitored, and should never be treated as metastases (32).

### Brain Metastases

Brain metastases are a rare occurrence in patients with testicular cancer. Only 2 to 3% of all patients will be diagnosed with metastatic lesions in the brain. The modality of choice in the diagnostic algorithm should be MRI, due to higher sensitivity and specificity in contrast to the CT. Using the full range of MRI capacities, we can differentiate the solid metastatic lesion from the cystic lesion or non-tumor pathological changes such as ischemia and inflammation (33). Brain metastases were found in 4 of 368 patients in a study using MRI imaging. Despite the non-uniform presentation, all patients had evidence of hemorrhagic element (33). A gradient-echo called hemosequence or 3D gradient-echo susceptibility weighted imaging (SWI) sequence detects the presence of the hemoglobin degradation products, thus allowing to identify a microhemorrhage. With the combination of these MRI features, the radiologist can narrow his focus in the assessment of images and could predict the micro metastases not seen on regular MRI. Further studies are required to confirm this thesis (33). However, conventional MRI is recommended by European society for medical oncology (ESMO), National Comprehensive Cancer Network (NCCN) in IGCCCG poor risk patients within the initial staging procedures (24, 34).

## The Role of FDG-PET/CT Scanning

Fluor-deoxy glucose (FDG) positron emission tomography/computer tomography (PET/CT) is generally not recommended as a standard diagnostic modality for primary staging according to the ESMO Clinical Practice Guidelines for GCT diagnosis (24). Its utility in the post-chemotherapy setting to assess the residual disease in patients with seminoma is still debated.

### Post-Chemotherapy FDG-PET/CT in the Management of Metastatic Seminoma

Viable cancer cells can be found in 12–30% patients after the first-line chemotherapy for the advanced stage of seminoma with a residual retroperitoneal mass larger than 3 cm. A residual mass smaller than 3 cm has been associated with <10% viable cancer cells found during the pathological examination (36P). Owing to the desmoplastic reaction between the residual mass and surrounding tissue, the post-chemotherapy RPLND is often extremely demanding, and surgery-associated morbidity is high. The role of PET/CT scanning and its predictive role in the management of metastatic seminoma have been widely discussed in the past years.

A negative FDG-PET/CT performed no earlier than six weeks following the completion of chemotherapy is highly reassuring by the virtue of a consistently reported high negative predictive value. A negative outcome of the PET/CT examination done in patients four to six weeks after the last chemotherapy has a reliable negative predictive value and no other treatment is required (35–40). In the case of a positive predictive value, there is no uniform point of view on the matter. Various post-chemotherapy changes, such as granulomatous-inflammatory changes and necrosis, may affect the PET/CT examination and cause false positive readings. Therefore, several studies which were carried out provided inconsistent results. In 2011, a retrospective validation of the multicentric SEMPET trial was performed to evaluate a clinical value of FDG-PET/CT in 125 patients with post-chemotherapy residual lesions (>3 cm versus ≤3 cm). Authors have observed the sensitivity, specificity, negative predictive value, and positive predictive value of 79% (95% CI = 52 to 92%), 81% (95% CI = 90 to 89%), 94% (95% CI = 84 to 98%), and 50% (95% CI = 31 to 69%), respectively, for the residual disease ≥3 cm; 43% (95% CI = 16 to 75%), 83% (95% CI = 70 to 91%), 91% (95% CI = 78 to 96%), and 27% (95% CI = 10 to 57%), respectively, for the residual disease <3 cm in size (**Table 1**) (30). In 2014, a meta-analysis of nine studies that included 375 scans of heterogeneous group of patients with residual lesions <3 cm and >3 cm, provided information on the pooled positive predictive value of 58% (95% CI: 48–68%) and negative predictive value of 94% (95% CI: 90–96%), specificity of 86% (95% CI: 81–89%), and sensitivity of 78% (95% CI: 67–87%) (**Table 2**). In this study, authors concluded that PET/CT scanning had better diagnostic accuracy for residual lesions bigger than 3 cm in comparison to those with residual lesions smaller than 3 cm (41). In the latest retrospective study done in 2018, the pooled positive predictive value showed the poorest performance. In the cohort of 90 patients with FDG/PET positive residual retroperitoneal tumor mass, the PPV was only 23% (**Table 3**). The previous smaller series included only few PET-positive patients (ranging from 8 to 33 patients) (42). The group size and strict criteria such as unequivocal PET positivity and elevated SUV of ≥4 might be the reason why the PPV is, in comparison with other studies, so low. Furthermore, the PET scans were not centrally reassessed and were done by local investigators which might have introduced an interobserver bias (42).

**Table 1:** Performance of PET imaging compared to CT in post-chemotherapy management of seminoma (30).

Residual disease (cm)	Seminoma post chemotherapy					
	PET			Radiologic monitoringCT		
	<3	≥3	All lesions	<3	≥3	All lesions
**Sensitivity**	43%	74%	67%	0%^a^	100%^a^	67%
**Specificity**	83%	70%	82%	100%^a^	0%^a^	44%
**PPV**	27%	37%	42%	87%	NA	87%
**NPV**	96%	92%	93%	NA	19%	19%

a Per definition: PET, positron emission tomography; CT, computed tomography; CI, confidence interval; NA, not applicable.

**Table 2:** Performance of PET imaging in post-chemotherapy management of seminoma (41).

	Seminoma post chemotherapy	
	Positron emission tomography (PET)	
		95% CI
**Sensitivity**	78%	67–87%
**Specificity**	86%	81–89%
**PPV**	58%	48–68%
**NPV**	94%	90–96%

**Table 3:** Performance of PET imaging in post-chemotherapy management of seminoma (42).

	Seminoma post chemotherapy								
	Positron emission tomography (PET) Cathomas et al. (42)								
	All patients	PET equivocal	PET definite	PET ≤ 6 weeks	PET > 6 weeks	PET SUV ≥ 4	PET SUV > 4	Lesion <3 cm	Lesion ≥3 cm
**No. of patients**	90	28	62	37	53	34	39	8	82
**PPV**	23%	11%	29%	29%	19%	32%	21%	38%	22%

The European consensus among experts for management of GCTs concluded that low probability of vital seminoma in residual masses <3 cm in the largest diameter renders the performance of PET/CT scanning as insufficient; thus, it should not be used in this clinical scenario (43). These patients should be observed with CT or magnetic resonance (MRI) up to 5 years (4). For patients with residual lesions >3 cm in the retroperitoneum, PET/CT maintains a high negative predictive value of 94–96%; therefore, the negative result of PET/CT should be used as a strong argument for the observation of a residual mass without the further treatment (42). However, while positive value of 23–50% for the residual disease >3 cm may provide an argument for PC-RPLND, submitting a patient for surgery may often lead to extreme postoperative morbidity (42). Acquiring a biopsy in such clinical scenario is not feasible due to high risk of false negativity from a single (or even multiple) core-cut biopsy. The viable cancer may often be size-limited or even microscopic; therefore, the biopsy should not be generally recommended in our opinion. Resection of the residual retroperitoneal mass preferable to biopsy is also recommended by the European consensus conference on diagnosis and treatment of germ cell cancer (44). Because of the extremely challenging management of PET-positive post-chemotherapy residual mass in seminoma, we recommend one follow-up PET-CT scan within 3 months. If the persisting PET positive tumor mass is considered relatively easily resectable by an expert urologic surgeon, the PC-RPLND should be done. On the other hand, if the resection would bring an extensive morbidity, we recommend further follow-up CT scans with salvage chemotherapy initiation upon the unequivocal finding of growing tumor mass.

### Post-Chemotherapy FDG-PET/CT in the Management of Metastatic Non-Seminoma

The benefit of FDG/PET utility in non-seminoma patients has been a question for discussion. There are reports that favor the use of FDG PET in NS patients, but also those rejecting the relevant benefit (45–47). A study that focused on NS patients assessed 85 residual lesions of which 32 (38%) showed an increased tracer uptake. This study has shown a sensitivity of 59% (95% CI: 44–73%), specificity of 92% (95% CI: 78–98%) NPV of 62% (95% CI: 48–75%), and PPV 91% (95%CI: 75–98%), thus showing the possible superiority of PPV rather than NPV in NS (**Table 4**) (29). This may be largely based on the frequent presence of a teratoma, which, like necrosis or scar, has a low FDG uptake at the conventional static PET scanning at 60 min after injection (48). The visual interpretation and SUV_lean_ were without difference among the evaluated lesions in 21 patients. In further analysis researchers detected statistically significant differences in the kinetic rate constants K1 and K between mature teratoma and necrosis or scar (K1, 0.113 ml/min/g ± 0.026 vs 0.036 ml/min/g ± 0.005 (P <.05); K, 0.005 ml/min/g ± 0.003 vs 0.0008 ml/min/g ± 0.0001 (P <.05). Therefore, FDG PET with kinetic analysis would be a promising method in evaluating residual lesion in non-seminoma patients in post chemotherapy settings. Currently, the inability to distinguish teratoma from necrosis and scar tissues is the main disadvantage of FDG PET (21, 29, 49, 50). For this reason, patients with the residual mass larger than 1 cm post chemotherapy must undergo an RPLND. Diagnostic utility of PET CT in case of the residual mass progressing in size on conventional CT with rising serum markers is without further benefit (51). Interesting findings were provided by a study assessing the intuition of a urologic surgical expert in distinguishing the outcome of post-chemotherapy RPLND. In this study, urologic surgical experts have done a complex analysis of patient history, orchiectomy histology, post-chemotherapy CT scans, and tumor markers on 53 patients who underwent RPLND. The intuition matched the final pathology in 70% of all cases. Teratoma and necrosis were correctly predicted in 79 and 54% of cases, respectively (52).

**Table 4:** Performance of PET imaging compared to MR/CT in post-chemotherapy management of NSGCT (29).

NSGCT post chemotherapy				
	PET		Radiologic monitoring (MR/CT)	
		95% CI		95% CI
**Sensitivity**	59%	44–73%	55%	40–69%
**Specificity**	92%	78–98%	86%	79–100%
**PPV**	91%	75–98%	84%	67–95%
**NPV**	62%	48–75%	58%	44–72%

In the light of currently available evidence, we do not recommend the use of PET/CT scanning in the post-chemotherapy imaging of non-seminoma. We believe such imaging may be often misleading and should not support a decision making for PC-RPLND. While always PET negative residual teratoma in lymph nodes >1 cm must be surgically resected in all cases, the clear survival benefit for patients undergoing PC-RPLND underlines the necessity of this procedure regardless of PET-CT findings. Ten to twenty percent of patients with lymph nodes larger than 1 cm harbor viable non-seminomatous cancer (53, 54). The survival benefit for PC-RPLND in this scenario has been repeatedly shown (53, 55). For instance, we believe that routine implementation of PC-RPLND in non-seminoma patients at our institution in 2008 contributed to the significantly improved outcomes for overall survival in series of 426 patients treated before and after 2008 (HR = 0.44, 95% CI 0.30–0.65; P = 0.0003) (56).

### FDG-PET/CT in the Management of Stage I Non-Seminoma

According to the study evaluating 50 patients, FDG-PET staging was equivalent to the CT staging (22). A large prospective trial concluded that the PET scanning is not sufficiently sensitive for identifying patients at low risk of relapse. This study enrolled 116 patients with the stage I disease. Current evidence does not support the routine use of FDG PET/CT in clinical management of patients with stage I non-seminoma (21, 22, 57).

## Adverse Health Risks Resulting From The Radiation Exposure of imaging Studies

The goal of modern CT imaging is to reduce the radiation dose to the minimum. The risks resulting from the radiation exposure in GCT patients often raises a concern among clinicians. GCT patients are young males who may need numerous imaging examinations during the treatment or they require surveillance for an extended time period. Several studies assessed the risk of secondary malignant neoplasms in the testicular cancer survivors. A large study analyzed 40,576 survivors with the average follow-up time of 11.3 years, finding the abdominal-pelvic malignancy rate of 30 malignancies per 10,000 person-years observation and thus statistically significantly increased long-term risk of second solid tumors (58). However, this study assessed the risk of secondary malignancies in all survivors undergoing surveillance or treatment. Thirty-three percent of patients received radiotherapy, and 99% patients received either radiotherapy or chemotherapy. Analyzed data showed an increased risk of solid cancers among patients treated with radiotherapy alone [relative risk (RR) = 2.0, 95% CI =1.9 to 2.2], chemotherapy alone (RR = 1.8, 95% CI = 1.3 to 2.5), and combined (RR = 2.9, 95% CI = 1.9 to 4.2). Due to the heterogeneity of the analyzed data, it is impossible to distinguish the late toxic effects of diagnostic scanning based on these results. Studies assessing the risk of second cancer from the radiation exposure of CT scans during the active surveillance should be performed to answer the concerning question of CT-induced secondary cancers (58). A study focusing on a group of 414 patients with stage I NSGCT directly compared two CT scans regimen to the five CT scan regimen during the active surveillance. Two hundred and forty-seven patients were examined with two CT scans at 3 and 12 months, and 167 patients received five scans at 3, 6, 12, and 24 months, with a median follow up of 40 months. The number of relapses observed in both groups were 37 (15%) in the two-scan arm and 33 (20%) in the five-scan arm. The relapse-free rates of 84.7% (95% CI = 79.5 to 88.8%) in the two-scan arm and 79.6% (95% CI = 72.6 to 85.1%) in the five scan arm were not statistically significantly different (P = 0.21). Patients with intermediate prognosis, who experienced relapse, were present in both groups, two (0.8%) in the two-scan arm and one (0.6%) in the five-scan arm. None of the patients who relapsed had poor prognosis, and no deaths were reported (59). A study by van Walraven et al. analyzed 2569 survivors, with the average follow-up time of 11.2 years. Authors did not include patients previously treated for other cancer, who had RPLND or radiotherapy, while 31% of patients were treated with chemotherapy. Patients underwent a median of 10 CT scans (interquartile range 4–18) during their 5-year follow-up. The median radiation dose was 110 mSv (interquartile range 44–190). Fourteen men were diagnosed with abdominal-pelvic secondary malignancy, which represents five per 10,000 patient-years observation. The hazard ratio for secondary malignancy per 10 mSv increase was 0.99 (95% CI, 0.95–1.04). In the multivariable analysis, the radiation exposure did not present a significant interaction between chemotherapy or age (60). While the risk of second cancer resulting from diagnostic radiation exposure appears to be insignificant after a median of 11.2 years, the longer follow-up should provide more insights on such risk after >2 decades since the initial diagnosis (52, 60).

MRI could be the modality of choice in the long-term follow up, due to its comparable diagnostic benefit and no risk of the radiation exposure. To this date, the main reasons for not using MRI routinely are not medical but rather economic and organizational. Patients undergoing the planned follow-up have a clear interval of control, and therefore, it would be possible to plan the MRI examinations in advance. The benefit of MRI could exceed the cost and toxicity burden of the examination. However, unknown long-term risk of contrast enhanced MRI should be assessed in clinical trials to further increase our knowledge for the diagnostic assessment with the best reproducibility and the lowest long-term toxicity.

## Conclusion

Testicular GCTs are the only universally curable solid malignancies. Proper management of patients with germ cell tumors requires a multidisciplinary approach based on the collaboration of a radiodiagnostician, urologist/surgeon, clinical oncologist, and radiation oncologist.

Radiology plays an important role in the diagnostic–therapeutic process. Imaging methods are essential in the initial diagnosis, active surveillance, post-chemotherapy management, and post-treatment follow-up. The radiographic imaging is an important part in the decision making within the therapeutic algorithm, planning of surgical procedures, and diagnosing of relapse. Ultimately, a precise imaging and correct radiologic assessment and interpretation in line with the clinical presentation are imperative for an optimal management of GCTs. GCTs require an expert multidisciplinary approach in a high-volume center to prevent the errors in the management leading to the unnecessary loss of lives in young curable patients.

## Author Contributions

MMa—researching data, article drafting, approving final version. KR—researching data, article editing, approving final version. IU—researching data, article editing, approving final version. MMe—researching data, article editing, approving final version. MC—researching data, article drafting, article editing, approving final version. All authors contributed to the article and approved the submitted version.,

## Funding

This work was supported by the Slovak Research and Development Agency, [No. APVV-15-0086, No APVV-19-0411 and grant VEGA 1/0327/19.

## Conflict of Interest

The authors declare that the research was conducted in the absence of any commercial or financial relationships that could be construed as a potential conflict of interest.
